# Treatment of non-systemic Sjögren's syndrome: Potential prevention of systematization with immunosuppressant agent/biotherapy

**DOI:** 10.1016/j.jtauto.2024.100238

**Published:** 2024-03-03

**Authors:** Aude Belbézier, Thi Thu Thuy Nguyen, Mélanie Arnaud, Bruna Ducotterd, Marie Vangout, Alban Deroux, Catherine Mansard, Françoise Sarrot-Reynauld, Laurence Bouillet

**Affiliations:** a Clinique Universitaire de Médecine Interne, Department of Internal Medicine, Grenoble University Hospital, F-38000, Grenoble, France

**Keywords:** Sjögren's syndrome, Biotherapy, Immunosuppressant agent

## Abstract

Sjögren's syndrome (SS) is a systemic autoimmune pathology manifested mainly by a dry syndrome, intense asthenia and arthromyalgia. Systemic manifestations may also occur. Since 2019, immunosuppressant agents (IS) or biotherapies are recommended only for patients with systemic involvement. However, before 2019, in some cases, paucisymptomatic patients had been treated with IS/biotherapies, often off-label. **Objective**: We propose to evaluate the benefit and safety of using IS/biotherapy in patients with SS without systemic involvement. **Methods:** We retrospectively collected the clinical records of all patients with SS diagnosed according to ACR/EULAR diagnostic criteria followed up between January 1980 and October 2023 at Grenoble University Hospital (France). **Results:** Eighty-three patients were included: 64 with an initially non-systemic form. Of these patients with an initially non-systemic form, 24 were treated with IS/biotherapy. None of them developed secondary systematization, whereas 11 out of 40 patients in the untreated group did (p < 0.05). On the other hand, IS/biotherapy did not appear to improve dry syndrome. There were no serious adverse events. **Conclusion:** Early introduction of an IS/biotherapy treatment appears to provide a benefit for the patient without side effects.

## Highlights

• SS is a chronic inflammatory disease that presents systemic complications either immediately or secondarily, with the risk increasing over time..
• Immunosuppressive treatment or biotherapy can prevent the onset of systemic manifestations..
• Pilocarpine does not appear to improve dry syndrome or its complications over the long term..

## 1 Introduction

Sjögren's syndrome (SS) is a systemic autoimmune disorder with a tropism for exocrine glandular epithelia [1]. Its main symptoms are dry eyes, dry mouth, gynecological dryness and dry skin [1]. Associated with this dry syndrome, there is frequently intense asthenia and arthromyalgia [1]. Systemic manifestations may also occur, mainly affecting lungs, lymph nodes and nerves [1].

This disease is far from rare: its prevalence is estimated between 0.6 and 1.5% of the general population. It mainly affects women (sex ratio F/M: 9/1), with a peak prevalence in the fifth decade [1].

Diagnosis is provided by the ACR/EULAR classification criteria of SS published in 2016 [1]. It is primarily based on the presence of an objectified dry syndrome associated with the demonstration of autoimmunity directed against the glandular epithelium, with the presence of lymphocytes within the accessory salivary glands or the presence of serum autoantibodies directed against the SSA protein [1].

Treatment depends on the type of organ affected.

When the symptomatic triad of dry syndrome, arthromyalgia-like pain and asthenia is present, purely symptomatic management is recommended, according to the EULAR recommendations [2] published in 2020 and the 'Protocole National de Diagnostic et de Soins' (PNDS) published in 2022 [3]. It relies on topical or systemic treatments to reduce dry syndrome, notably with muscarinic agonists [2,3] such as pilocarpine [[3], [4], [5], [6]], and pain, notably with the use of non-steroidal anti-inflammatory drugs (NSAID) or hydroxychloroquine (HCQ) [2,3].

In the case of systemic manifestations, immunosuppressive treatments (IS) or biotherapy such as belimumab [7], rituximab [8] and abatacept [9] are generally recommended.

Prior to the recommendations issued by the PNDS [3], IS or biotherapies were sometimes used in patients presenting the pathognomonic symptomatic triad with dry syndrome resistant to topical treatments, or presenting arthromyalgia with significant functional repercussions. Those treatments were often introduced to prevent secondary systematization of the disease, although no studies have demonstrated this hypothesis.

Moreover, the use of muscarinic agonists, which are recommended for dry syndrome resistant to topical treatment, is often limited by their tolerance.

We propose to evaluate the benefit and the safety of using IS agent or biotherapy in patients with Sjögren's syndrome without systemic involvement in Grenoble University Hospital. We will also evaluate the benefit of a muscarinic agonist, pilocarpine (the only agonist with marketing authorization in France), in dry syndrome in our population of patients followed in internal medicine for Sjögren's disease with or without systemic form.

## 2 Methods

### 2.1 Patients’ selection

We retrospectively collected clinical files from all patients with SS diagnosed according to ACR/EULAR diagnosis criteria followed between January 1980 and October 2023 in the Grenoble University Hospital (France).

Exclusion criteria included follow-up of less than 6 months, absence of a dry syndrome or participation in a pilot study (due to lack of knowledge of the therapy delivered to the patient). The local medical ethics committee approved the study the 9/7/23 (named E2TMG3S) and oral informed consent was obtained from each patient.

### 2.2 Data analysis

Clinical data were extracted from medical records that we collected periodically in the internal medicine department at Grenoble University Hospital. We reviewed all clinical records of patients hospitalized or followed up in consultation in internal medicine to select all patients with ≥6 months of follow-up.

Systemic SS was defined according to the PNDS criteria [3] as a form not limited to the 'dry syndrome-arthromyalgia-asthenia' triad. Systemic manifestations could be hematological (cytopenia), pulmonary (diffuse interstitial lung disease), neurological (central or peripheral with abnormalities detected on imaging, lumbar puncture or electromyogram), articular -with the presence of arthritis-, renal, cutaneous -with the presence of vasculitis or purpura-, muscular -with the presence of myositis-.

ESSDAI ≥1 was not synonymous with systemic disease, given the presence of glandular, lymph node and joint manifestations such as arthralgia in the ESSDAI score.

Dry syndrome was subjectively assessed by the physician at the follow-up consultation.

A major improvement corresponds to a regression of the dry syndrome of more than 50%, a moderate regression to less than 50%.

IS included leflunomide, cyclophosphamide, mycophenolate mofetil, azathioprine and methotrexate.

The biotherapies used were rituximab, belimumab, abatacept and tocilizumab.

### 2.3 Statistical analysis

All statistical tests were performed by using GraphPad PRISM 5.0 (GraphPad Software, Inc, La Jolla, Calif).

Continuous variables were presented as medians and ranges. Non-parametric Wilcoxon test was used for the comparison of two groups. One-way analysis of variance followed by Bonferroni correction was used to compare more than two groups.

Categorical variables were presented with counts and percentages and were compared using the Chi square test (if number of patient was >5 patients) or Fisher test.

Risk of systematization was compared between groups using survival analysis with log rank test. All tests were two-sided. All tests were considered significant when p < 0.05.

## 3 Results

**Fig. 1:**
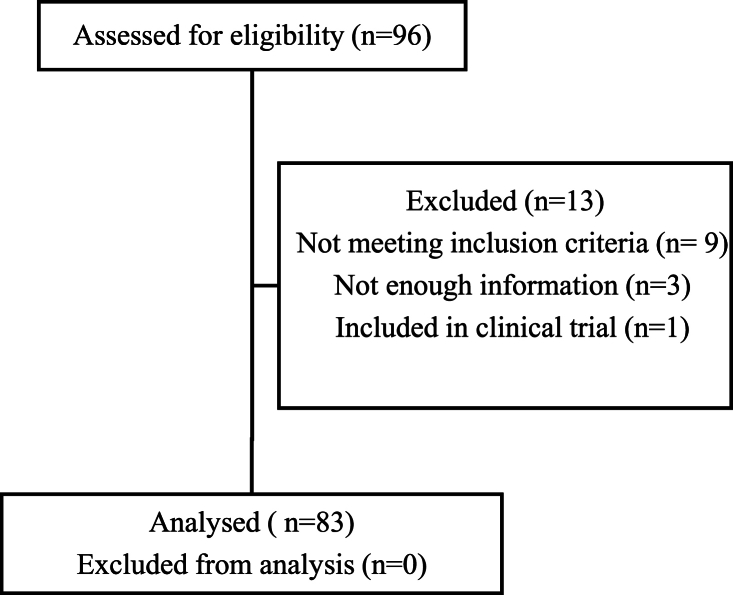
**Flow diagram.** Of a cohort of 96 patients, 83 patients were included with a confirmed Sjögren's syndrome.

Ninety-six patients were eligible to participate in this study. Thirteen were excluded: 10 because they did not meet the inclusion criteria (3 not meeting ACR/EULAR 2016 criteria [2], 5 did not complai of dry syndrome, 1 with less than 6 months follow-up, 1 included in a clinical trial) and 3 due to lack of information. Eighty-three subjects with SS were recruited for this study (Fig. 1). The clinical characteristics of the patients are presented in Table 1. Of all patients, 50 received one line of immunomodulatory treatment (IS/Biotherapy or HCQ), 37 two lines, 20 three lines, 7 four lines and 2 five lines of treatment (Table 2). Thirty nine patients received HCQ (30 with initially non-systematized SS form and 9 with initially systematized SS form).

**Table 1:** Characteristics of SS patients.

Number of patients (n)	83
Age at diagnosis, mean ± SD (years)	56.0 ± 14.3
Female, n (%)	73 (88.0)
Disease duration, mean ± SD (year)	11.3 ± 8.8
Time between onset and diagnosis, mean ± SD (year)	5.0 ± 7.0
Clinical manifestation, n (%)	
Parotid gland enlargement, n (%)	2 (2.4)
Extraglandular manifestation, n (%)	43 (51.8)
Polyarthralgia, n (%)	20 (24.1)
Arthritis, n (%)	8 (9.6)
Cutaneous vasculitis, n (%)	4 (4.8)
Raynaud's phenomenon, n (%)	18 (21.7)
Pulmonary involvement, n (%)	13 (15.9)
Neurological involvement, n (%)	9 (10.8)
Renal involvement, n (%)	3 (3.6)
Autoimmune cytopenia, n (%)	3 (3.6)
Myositis, n (%)	3 (3.6)
Lymphadenopathy	11 (13.4)
Biological parameters	
IgM-RF*, n (%)	24 (33.8)
Hypocomplementemia**, n (%)	13 (16.9)
Focus score***, n (%)	1.6 ± 1.0
Complication	
Cryoglobulinemia****, n (%)	6 (13.3)
Associated lymphoma, n (%)	3 (3.7)
Systemic treatment	55 (67.4)
Hydroxychloquine, n (%)	39 (47)
Immunosuppressant agent/biotherapy, n (%)	44 (53)
Pharmacological stimulation	
Pilocarpine therapy, n (%)	16 (19.2)
Anetholtrithione, n (%)	20 (24.1)

Data presented as mean ± SD or number (n) and percentage (%). IgM-RF: immunoglobulin M-Rheumatoid Factor. *data missing for 12 patients, **data missing for 6 patients, *** data missing for 9 patients, **** data missing for 38 patients.

**Table 2:** Immunotherapy on patients with or without initially systemic SS.

Variables (n)	Initially systemic SS (19)	Initially non-systemic SS (64)
Treatment, n (%)	16 (84)	24 (37.5)
Azathioprine, n (%)	3 (15.8)	1 (18.8)
Treatment time, mean ± SD (month)	86.5 ± 79.6	75
Improvement, n (%)	2 (66.7)	0 (0)
Time before improvement, mean ± SD (month)	21 ± 21.2	NA
Stop for poor tolerance, n (%)	1 (33.3)	0 (0)
Dry syndrome improvement, n (%)	1 (33.3)	0 (0)
Methotrexate, n (%)	5 (26.3)	16 (25)
Treatment time, mean ± SD (month)	47.8 ± 67.8	21.5 ± 23
Improvement, n (%)	3 (60)	12 (75)
Time before improvement, mean ± SD (month)	5 ± 1,4	4.2 ± 1.4
Stop for poor tolerance, n (%)	2 (40)	4 (25)
Dry syndrome improvement, n (%)	1 (20)	3 (20)
Leflunomide, n (%)	1 (5.2)	0 (0)
Treatment time, mean ± SD (month)	0.90	NA
Improvement, n (%)	0	NA
Time before improvement, mean ± SD (month)	NA	NA
Stop for poor tolerance, n (%)	0	NA
Dry syndrome improvement, n (%)	0	NA
Mycophenolate Mofetil, n (%)	3 (15.8)	0 (0)
Treatment time, mean ± SD (month)	27.9 (9.1)	NA
Improvement, n (%)	2 (68.7)	NA
Time before improvement, mean ± SD (month)	4.5 *	NA
Stop for poor tolerance, n (%)	0 (0)	NA
Dry syndrome improvement, n (%)	1 (33.3)	NA
Cyclophosphamide, n (%)	2 (10.5)	0 (0)
Treatment time, mean ± SD (month)	3.7 ± 3.6	NA
Improvement, n (%)	2 (100)	NA
Time before improvement, mean ± SD (month)	0.5 ± 0.7	NA
Stop for poor tolerance, n (%)	1 (50)	NA
Dry syndrome improvement, n (%)	0 (0)	NA
Abatacept, n (%)	1 (5.2)	10 (15.6)
Treatment time, mean ± SD (month)	14	4.7 ± 29.0
Improvement, n (%)	0 (0)	6 (60)
Time before improvement, mean ± SD (month)	NA	4.2 ± 2.2
Stop for poor tolerance, n (%)	0	1 (10)
Dry syndrome improvement, n (%)	0	4 (44)
Rituximab, n (%)	6 (31.2)	10 (15.6)
Treatment time, mean ± SD (month)	29.4 ± 30.9	21.0 ± 25.9
Improvement, n (%)	5 (50)	7 (35)
Time before improvement, mean ± SD (month)	4.1 ± 3.1	3.6 ± 2
Stop for poor tolerance, n (%)	1 (20)	1 (10)
Dry syndrome improvement, n (%)	3 (50)	3 (30)
Belimumab, n (%)	2 (10.5)	1 (1.6)
Treatment time, mean ± SD (month)	39.7 ± 7.8	7
Improvement, n (%)	2 (100)	0 (0)
Time before improvement, mean ± SD (month)	8.5 ± 3.5	NA
Stop for poor tolerance, n (%)	0 (0)	0 (0)
Dry syndrome improvement, n (%)	0 (0)	0 (0)
Tocilizumab, n (%)	0 (0)	1 (1.6)
Treatment time, mean ± SD (month)	NA	9.0
Improvement, n (%)	NA	0 (0)
Time before improvement, mean ± SD (month)	NA	NA
Stop for poor tolerance, n (%)	NA	1 (100)
Dry syndrome improvement, n (%)	NA	0 (0)
Number of treatment lines		
1st line, n (%)	11 (57.9)	39 (60.9)
2nd line, n (%)	12 (63.2)	25 (39.0)
3rd line, n (%)	7 (36.8)	13 (20.3)
4th line, n (%)	1 (5.2)	6 (9.3)
5th line, n (%)	0 (0)	2 (3.1)
Manifestation improving outside dry syndrome	9 (50)	9 (69)**
Arthritis, n (%)	5 (71.4)	7 (65)
Arhtralgia, n (%)	3 (37)	8 (88)
Other, n (%)	0 (0)	3 (30)
Neurological, n (%)	3 (66.7)	2 (40)
Pulmonary, n (%)	3 (33.3)	1 (25)
Kidney, n (%)	1 (50)	NA
Cutaneous, n (%)	2 (66.7)	NA

Data presented as mean ± SD or number (n) and percentage (%). NA: not applicable; *data missing for 1 patient; ** Data missing for 1 patient.

### 3.1 Outcome of patients with non-systemic SS with or without IS/Biotherapy

Sixty-four patients had a non-systemic disease at the start of follow-up in the internal medicine department. Of these, 24 were rapidly treated with IS agents or biotherapy (Table 2). Thirty patients received HCQ (15 in the IS/biotherapy group, 15 in the untreated group). Of the patient receiving HCQ, three showed improvement in dry syndrome and thirteen showed improvement in arthralgia.

**Fig. 2:**
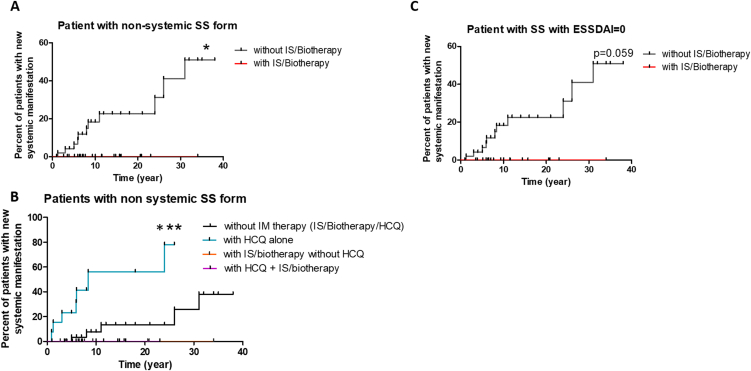
**Percentage of onset of systematic manifestations over time in patients followed up for SS** with a non-systemic form at baseline (A, B), with ESSDAI 0 (C), treated with immunomodulatory treatment (IS/biotherapy (A, B, C) and/or HCQ (B)). HCQ: Hydroxychloroquine, IS Immunosuppressant. *: p < 0.05; ***: p < 0.001.

Patients with a non-systemic form who received IS/biotherapy developed significantly less secondary systematization with increasing duration of follow-up (Fig. 2A). In fact, none of the IS/biotherapy treated patients developed secondary systematization during follow-up.

If we looked at the risk of systematization by separating patients with non-systemic SS according to the type of immunomodulatory treatment received (immunosupressant/biotherapy, HCQ or both combined), treatment with HCQ alone did not prevent systematization (Fig. 2B) (p < 0.001).

Finally, when we looked at the risk of systematization in patients with ESSDAI = 0, i.e. 59 patients (21 treated and 38 untreated with IS/biotherapy), the risk of systematization was greater in untreated versus treated patients, but not significantly so (p = 0.059) (Fig. 2C).

Finally, we wondered whether patients with secondary systematization presented a particular profile compared to those without (Table 3). Age at the diagnosis (p < 0.05), the length of follow up (11 years vs. 19 years in patients without and with secondary systematization respectively (p < 0.01)), and systemic involvement were different between the two groups.

**Table 3:** Difference between patients with non-systemic SS form, secondary systemic SS form or initial systemic SS form.

Variable (n)	Initially non-systematized SS form		Initially systematized SS form (19)	p value
	With no systematization (53)	Secondary systematized form (11)		
Age at diagnosis, mean ± SD (years)	58 ± 14.3	45.2 ± 16.6	55.7 ± 9.5	***0.0170***
Female, n (%)	49 (92.5)	10 (90.9)	14 (73.7)	0.0928
Disease duration, mean ± SD (year)	11 ± 7.3	19.3 ± 13.1	7.6 ± 4.8	***0.026***
Time between onset and diagnosis, mean ± SD (year)	5.3 ± 7.2	6.7 ± 9.1	2.9 ± 4.1	***0.0012***
Clinical manifestation				
Parotid gland enlargement, n (%)	1 (1.9)	1 (9.1)	0 (0)	0.27
Extraglandular manifestation, n (%)	13 (24.5)	11 (100)	19 (100)	***< 0.0001***
Polyarthralgia, n (%)	13 (24.5)	2 (18.2)	5 (26.3)	0.875
Arthritis, n (%)	0 (0)	1 (9.1)	7 (36.8)	***< 0.0001***
Cutaneous vasculitis, n (%)	0 (0)	1 (9.1)	3 (15.7)	***0.0174***
Raynaud's phenomenon, n (%)	9 (17)	4 (36.4)	5 (26.3)	0.3126
Pulmonary involvement, n (%)	0 (0)	4 (36.4)	9 (47.3)	***< 0.0001***
Neurological involvement, n (%)	0 (0)	4 (36.4)	5 (26.3)	***< 0.0001***
Renal involvement, n (%)	0 (0)	1 (9.1)	2 (10.5)	0.0627
Autoimmune cytopenia, n (%)	0 (0)	2 (18.2)	1 (5.3)	***0.0121***
Myositis, n (%)	0 (0)	0 (0)	3 (15.8)	***0.0053***
Lymphadenopathy, n (%)	7 (13.2)	1 (9.1)	3 (15.8)	0.8728
Biological parameters				
IgM-RF*, n (%)	12 (26.7)	3 (37.5)	9 (47.4)	0.2036
Hypocomplementemia**, n (%)	6 (12.5)	2 (18.2)	5 (27.8)	0.3341
Focus score***, mean ± SD	1.69 ± 1.03	1.55 ± 0.82	1.47 ± 0.83	0.48
Complication				
Cryoglobulinemia****, n (%)	3 (10)	1 (33.3)	2 (17)	0.9771
Associated lymphoma, n (%)	2 (3.8)	0 (0)	1 (5)	0.754
Systemic treatment, n (%)	33 (62.2)	6 (54.5)	16 (84)	0.1501
Hydroxychloquine, n (%)	24 (45.3)	6 (54.5)	9 (47)	0.8542
Immunosuppressant agent/Biotherapy, n (%)	24 (45.3)	5 (45.5)	14 (74)	0.4371
Leflunomide, n (%)	0 (0)	0 (0)	1 (5)	0.1818
Mycophenolate mofetil, n (%)	0 (0)	0 (0)	3 (16)	***0.0053***
Azathioprine, n (%)	1 (1.9)	0 (0)	3 (16)	***0.0381***
Methotrexate, n (%)	16 (30.2)	2 (18.2)	5 (26)	0.7119
Cyclophosphamide, n (%)	0 (0)	0 (0)	2 (11)	***0.0317***
Abatacept, n (%)	10 (18.9)	1 (9.1)	1 (5)	0.3029
Rituximab, n (%)	10 (18.9)	4 (36.4)	6 (32)	0.32
Belimumab, n (%)	1 (1.9)	0 (0)	2 (11)	0.1762
Tocilizumab, n (%)	1 (1.9)	0 (0)	0 (0)	0.7509
Pharmacological stimulation				
Pilocarpine therapy, n (%)	14 (26.4)	1 (9.1)	1 (5)	0.0877
Anetholtrithione, n (%)	14 (26.4)	3 (27.3)	3 (16)	0.6271

Data presented as mean ± SD or number (n) and percentage (%). IgM-RF: immunoglobulin M-Rheumatoid Factor. *data missing for 12 patients; **data missing for 6 patients; *** data missing for 9 patients; **** data missing for 38 patients.

### 3.2 Outcome of patients with systemic form with or without IS/Biotherapy

Of the 19 patients with systemic onset, 15 received IS/biotherapy. Four patients did not receive treatment despite a systemic form: 1 had a CNS form with white matter hypersignals, 1 had pulmonary involvement with bronchitis and bronchial dilatation, 1 had myositis which improved spontaneously, and 1 had a multivisceral form with hematological, neurological, articular and cutaneous involvement under investigation. HCQ treatment was given for a time to the patient with pulmonary involvement, but was ineffective.

When we looked at the risk of new systemic manifestations, there was no difference between the treated and untreated arms. Indeed, among the four untreated patients, only one presented a new systemic manifestation. This patient's initial multisystemic form worsened rapidly, with hematological and muscular involvement, before treatment with rituximab was initiated. Despite this treatment, she developed secondary cryoglobulinemia and died within 18 months of diagnosis.

In the treatment arm, one patient developed moderate proteinuria with an unlabeled glomerular profile after 11 years follow-up, despite well-managed treatment with rituximab. This proteinuria improved spontaneously.

### 3.3 Progression of dry syndrome with muscarinic agonist or with IS/Biotherapy

**Fig. 3:**
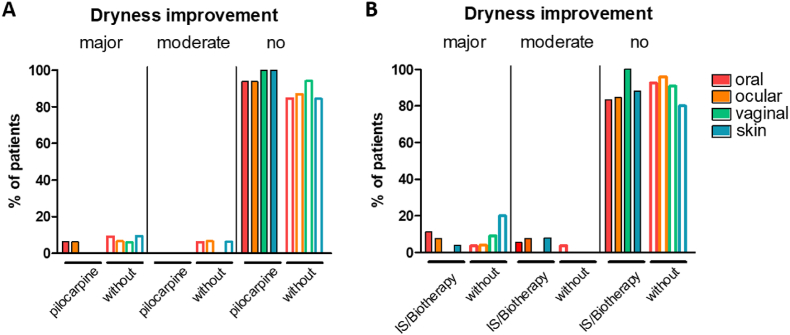
**Evolution of dry syndrome in patients with SS treated or not with pilocarpine (A) or IS/Biotherapy.** A major improvement corresponds to a regression of the dry syndrome of more than 50%, a moderate regression to a regression of less than 50%. IS: immunosuppressant agent.

We then studied the value of pilocarpine in improving dry syndrome in patients with SS. Sixteen patients received pilocarpine. Among them, two patients discontinued the treatment due to poor tolerance. When we look at the evolution of the dry syndrome, it did not differ between treated and untreated patients (Fig. 3A), with comparable dryness between the two groups and a comparable number of complications such as recurrent gingivo-stomatitis, recurrent parotiditis, tooth decay, blepharitis, conjunctivitis, keratitis, candidiasis (oral, genital). On the other hand, treated patients had fewer episodes of parotid swelling (Table 4).

**Table 4:** Characteristics of SS patients treated or not with pilocarpine.

Variable (n)	With pilocarpine (16)	Without pilocarpine (67)	p value
Age at diagnosis, mean ± SD (years)	54.9 ± 11.3	56.3 ± 14.9	0.7302
Female, n (%)	15 (93.8)	58 (86.5)	0.4278
Disease duration, mean ± SD (year)	14.8 ± 8.9	10.4 ± 8.6	0.0752
Parotid gland enlargement, n (%)	0 (0)	2 (3.0)	***0.0432***
Extraglandular manifestation, n (%)	6 (37.5)	37 (55.2)	0.2024
Glandular manifestation			
oral, n (%)	16 (100)	64 (95.5)	0.3886
ocular, n (%)	16 (100)	61 (91.0)	0.214
vaginal*, n (%)	9 (75)	20 (48.8)	0.1438
skin**, n (%)	6 (46.2)	31 (50)	0.8009
Focus score ***, mean ± SD	1.5 ± 0.74	1.64 ± 1.0	0.9194
IgM-RF****, n (%)	3 (21.4)	21 (36.8)	0.2747
Immunomodulatory agent, n (%)	11 (68.8)	45 (67.16)	0.90
Hydroxychloquine, n (%)	9 (56.25)	30 (44.78)	0.4087
Leflunomide, n (%)	1 (6.25)	0 (0)	0.4333
Mycophenolate mofetil, n (%)	0 (0)	3 (4.48)	0.9071
Azathioprine, n (%)	0 (0)	4 (5.97)	0.7247
Methotrexate, n (%)	3 (18.75)	20 (29.85)	0.5616
Cyclophosphamide, n (%)	0 (0)	2 (2.99)	1.00
Abatacept, n (%)	4 (25)	8 (11.94)	0.3477
Rituximab, n (%)	5 (31.25)	15 (22.39)	0.6749
Belimumab, n (%)	0 (0)	3 (44.78)	0.9071
Tocilizumab, n (%)	0 (0)	1 (1.49)	1.00
Dry syndrome improvement			
oral, n (%)	1 (6.3)	10 (15.6)	0.3578
ocular, n (%)	1 (6.3)	8 (13.1)	0.5107
vaginal*, n (%)	0 (0)	1 (5.9)	0.6765
skin, n (%)	0 (0)	5 (15.6)	0.6851
Dry syndrome complication, n (%)	7 (43.8)	30 (44.8)	0.9409

Data presented as mean ± SD or number (n) and percentage (%). *data missing for 20 patients; **data missing for 8 patients; *** data missing for 9 patients; **** data missing for 10 patients.

We also looked at the efficacy of immunosuppressive therapies or biotherapy on dry syndrome. Those therapies did not change the course of the dry syndrome either (Fig. 3B).

### 3.4 IS/biotherapy tolerance

IS/biotherapy treatments were often well tolerated (Table 2). The most frequent adverse events were digestive disorders on methotrexate and abatacept, arthralgia and asthenia after a course of rituximab. Two treatments were discontinued because of infections: leflunomide because of skin abscesses, and tocilizumab because of repeated non-serious infections. Two cases of significant hepatic cytolysis (but without any drop in PT) were reported: one on azathioprine, one on methotrexate.

## 4 Discussion

SS is a chronic inflammatory disease with a polymorphic presentation for which no treatment has been shown to be effective [[10], [11], [12], [13]]. Indeed, to date, no clinical trial has shown significant improvement in the disease under treatment. However, this information must be qualified by the fact that few molecules have been tested in Phase 3 trials (only hydroxychloroquine [14], rituximab [12,13,15], abatacept [11] and etanercept [16]). Nevertheless, even in case analysis, few treatments appear to be effective. Some teams have reported some efficacy of rituximab on glandular involvement [12,15,[17], [18], [19], [20]]. However, those results are debated, with other teams finding no evidence of efficacy [13,[21], [22], [23]]. This has led international specialists [2,3] to recommend purely symptomatic treatment for non-systemic disease, with immunosuppressive therapy or biotherapy reserved for the most severe forms, by extrapolation of data found for other autoimmune diseases, notably lupus [24].

It is therefore recommended that dry syndrome be treated first with topical agents, followed by muscarinic agonist if symptoms persist despite topical treatment [2]. We also wanted to examine the efficacy of long-term pilocarpine on dry syndrome. In France, only pilocarpine is authorized as a muscarinic agonist, although it is considered less well tolerated than cevimeline [25]. Pilocarpine has indeed demonstrated some efficacy in reducing oral and ocular dry syndrome [4,26,27], both subjectively and objectively at early stages (≤12 weeks), with an improvement in salivary flow and conjunctival inflammation (measured via conjunctival cellularity) [4]. However, its efficacy has never been tested at more than 12 weeks' follow-up. The lack of difference between the pilocarpine-treated and non-treated groups of SS patients can be explained by the absence of objective measures in this study, but also by the lack of long-term efficacy of pilocarpine. Pipje et al. studied the evolution of the dry syndrome over time in patients with SS [28]. They found a slight worsening of stimulated salivary flow and stimulated parotid glandular volume after 4 years of disease progression, but these were not associated with a worsening of the sensation of dry mouth or dry eyes [28]. Nor is there any change in unstimulated salivary flow or glandular volume. In our study, we found no reduction in the risk of dry syndrome complications such as recurrent gingivo-stomatitis, recurrent parotiditis, tooth decay, blepharitis, conjunctivitis, keratitis, candidiasis (oral, genital). This raises the question of the value of this molecule as a long-term treatment, all the more so as it is often poorly tolerated [25], reducing patient compliance. Further studies to investigate the long-term efficacy of pilocarpine in preventing complications of dry syndrome would also appear to be necessary.

Apart from symptomatic management of dry syndrome, it is recommended that patients with non-systemic SS have joint pain managed with NSAIDs, followed by HCQ if NSAIDs prove effective [2,3]. In our study, this treatment improved joint pain in 31.5% of cases. However, it did not prevent systematization. In our study, where patients were followed-up for an average of 11 years, we can see that patients with a non-systemic form at the outset had a secondary risk of systematization, which increased with time.

The risk of systematization of the disease is well known [29]**,** which has led various health authorities to recommend regular follow-up of patients with SS [2,3], and the introduction of treatment if systematization occurs. In our study, we can see that the introduction of IS or biotherapy in systemic forms is only effective in around 50% of cases. When introduced before systematization, it seems to prevent the appearance of new manifestations. This is in line with findings for other chronic dysimmune diseases such as Behcet's disease or interferonopathies, where failure to control inflammation is associated with an increased risk of disease worsening [30,31]**.** The value of treating all chronic inflammation is also increasingly debated, as the latter seems to favor both cardiovascular pathologies hitherto considered as atheromatous disease, and neuropsychiatric pathologies [[32], [33], [34]]**.** The question remains as to which molecule to use and how to control inflammation. In our study, apart from leflunomide or tocilizumab, any IS or biotherapy seemed to prevent systematization. This should be weighed against the fact that prescribing physicians regularly changed treatment (>5% of patients received more than 4 lines) when they judged the current treatment to be ineffective.

Dryness intensity does not seem to be a marker of systematization. In our study, it did not regress under immunosuppressive treatment. Those results are in line with other studies showing non-regression of dry syndrome under treatment **[**5, 21–31**],** both subjective and objective. As well, initial arthralgia do not appear to be a predictor of secondary systematization. In rheumatoid arthritis, the DAS28 not only assesses the number of swollen, painful joints, but also the subjective evaluation of the disease [35]**.** A composite score taking into account objective disease assessment (such as IgM-RF, complement), salivary flow and subjective assessment using a visual analog scale, as is the case for DAS28, could be an interesting tool.

This study has several limitations. The duration of follow-up between the different forms varies: it is longer in patients with a secondarily systematized form than in patients with a non-systematized form. This may raise the question of a potential bias when comparing the risk of systematization in patients with an initially non-systematic form between the IS/biotherapy-treated group and the untreated group. Nevertheless, the Log Rank test normally takes these differences in follow-up time into account, and we can see that as early as 10 years, there is a difference between treated and non-treated groups. Another limitation is that the data were studied retrospectively. In addition, evaluation of dry syndrome was based solely on subjective assessment by the physician. Another limitation inherent to registry studies is that the clinical findings depend on physician expertise.

In conclusion, SS is a chronic disease with the risk of immediate or secondary systematization. Immunosuppressive treatments or biotherapy might prevent secondary systematization but further randomized studies are needed.

## Funding statement

None.

## Disclosure statement

None.

## CRediT authorship contribution statement

**Aude Belbézier:** Writing – original draft, Validation, Methodology, Investigation, Formal analysis, Conceptualization. **Thi Thu Thuy Nguyen:** Data curation. **Mélanie Arnaud:** Methodology, Data curation. **Bruna Ducotterd:** Data curation. **Marie Vangout:** Validation, Investigation. **Alban Deroux:** Validation, Investigation. **Catherine Mansard:** Validation, Investigation. **Françoise Sarrot-Reynauld:** Writing – review & editing, Validation, Investigation. **Laurence Bouillet:** Writing – review & editing.

## Declaration of competing interest

The authors declare that they have no known competing financial interests or personal relationships that could have appeared to influence the work reported in this paper.

## Data Availability

The data that has been used is confidential.
